# 脑脊液外泌体亚群的分离及其蛋白质组学分析

**DOI:** 10.3724/SP.J.1123.2024.10014

**Published:** 2025-05-08

**Authors:** Xiaofei CHEN, Wei LIU, Wenjia ZHANG, Yanpeng LI, Zhihua WANG, Mingxia GAO, Xiangmin ZHANG

**Affiliations:** 1.复旦大学化学系, 上海 200438; 1. Department of Chemistry, Fudan University, Shanghai 200438, China; 2.上海市浦东医院, 上海 201399; 2. Shanghai Pudong Hospital, Shanghai 201399, China

**Keywords:** 外泌体, 外泌体亚群, 蛋白质组学, 脑脊液, 创伤性脑损伤, exosomes, exosome subtypes, proteomics, cerebrospinal fluid (CSF), traumatic brain injury (TBI)

## Abstract

外泌体是一类尺寸为30~200 nm、携带有蛋白质等生物活性物质的细胞外小囊泡,能够分为具有不同功能的亚群,对外泌体异质性的了解有助于研究其在生理和病理中的作用机制。脑脊液来源的外泌体作为疾病的生物标志物具有巨大潜力,但是相关蛋白质组学的研究报道较少,且缺乏对脑脊液外泌体亚群的研究。本研究发展了串联体积排阻色谱法用于分离脑脊液外泌体亚群,并结合纳升级液相色谱-捕集离子淌度飞行时间质谱联用技术(nanoLC-TIMS-TOF-MS),对创伤性脑损伤患者的外泌体亚群进行了蛋白质组学分析。结果表明,利用串联体积排阻色谱法分离得到4个不同尺寸的脑脊液外泌体亚群,不同亚群共鉴定到739个蛋白质,进一步的蛋白质组学分析结果表明外泌体亚群与细胞信号转导、凝血过程和免疫反应等生物过程密切相关。此外,研究结果揭示了脑脊液外泌体亚群的蛋白表达存在异质性,特别是大尺寸的亚群在不同样本间存在较大的差异。综上,本研究利用开发的串联体积排阻色谱方法分离得到脑脊液外泌体亚群,并从蛋白质组学的角度丰富了对创伤性脑损伤患者脑脊液外泌体的认知。

## Abstract (en)

Exosomes are small extracellular vesicles 30-200 nm in diameter that contain many bioactive macromolecules, including proteins, lipids, and nucleic acids; consequently, they play important roles in many physiological and pathological processes and are classified into various property-dependent subtypes. Research into exosome heterogeneity helps broaden our understanding of the physiological and pathological mechanisms associated with exosomes. Exosomes exist in many human biological fluids, with those derived from cerebrospinal fluid (CSF) regarded as potential disease biomarkers. Despite this, few studies have focused on their proteomics, and little research into CSF-derived exosome subtypes has been reported. Traumatic brain injury (TBI) is a major public health issue characterized by a large number of patients and complex pathological processes. While a comprehensive understanding of the pathophysiological processes that underpin TBI is essential for developing therapeutic interventions, proteomic studies into CSF-derived exosomes in patients with TBI are limited. Herein, we designed a tandem size-exclusion chromatography protocol for isolating and profiling the proteins of CSF-derived exosome subtypes from patients with TBI using nanoscale liquid chromatography and trapped-ion mobility spectrometry time-of-flight mass spectrometry (nanoLC-TIMS-TOF-MS). We first centrifuged the collected CSF to remove cells and cell debris, after which it was concentrated by ultrafiltration to increase the exosome concentration and remove small proteins and peptides. A mini-size exclusion chromatography (Mini-SEC) column was then used to separate the exosomes from large amounts of interfering proteins, after which high performance liquid-SEC (HPL-SEC) was used to further separate exosomes according to size. The entire extracellular-vesicle-subset separation and purification process takes approximately 1 h for a single CSF sample. Four differently sized exosome subtypes were successfully isolated and are referred to as S1, S2, S3, and S4 in order of descending size. The S1 subtype exhibited the highest exosome purity according to the particle-to-protein ratio. Multiple characterization methods, including transmission electron microscopy (TEM), Western blotting (WB), and nanoparticle tracking analysis (NTA), confirmed that the exosome subtypes had been successfully acquired. NanoLC-TIMS-TOF-MS, combined with database searching were then used to characterize the proteins. A total of 739 proteins were identified, of which 79% and 72% matched all proteins and the top 100 proteins in the Vesiclepedia database, respectively. Moreover, gene ontology analysis revealed that the identified proteins are mainly located in extracellular exosomes, and that the isolated exosome subtypes are closely related to multiple biological processes, including cell signaling, coagulation, and immune responses. Hierarchical cluster analysis revealed that samples from the same exosome subset are grouped first. Principal-component and Pearson’s correlation coefficient analyses revealed that the proteins expressed in the CSF-derived exosome subtypes are heterogeneous. Interestingly, the proteins identified in the S1 subtype varied greatly between samples, highlighting the potential applicability of this subtype to formulating precise therapeutic regimens for different patients. We also analyzed the highly expressed proteins in the exosome subtypes, which revealed that the enrichment pathway of the S1 subtype involves Vitamin B12 metabolism and the regulation of protein catabolic processes, while the specific enrichment pathway of the S2 subtype includes binding and ligand uptake by scavenger receptors, heme scavenging from plasma, and an inflammatory response. In contrast, the unique enrichment pathway of the S3 subtype contains complementary and coagulation cascades and acute-phase responses, while that of the S4 subtype includes post-translational protein phosphorylation. Furthermore, STRING-based protein-association analysis predicted multiple interactions among proteins in the various exosome subtypes. In conclusion, the developed tandem size-exclusion chromatography method was used to isolate cerebrospinal fluid exosome subtypes. This study enriches knowledge regarding cerebrospinal fluid exosomes in patients with TBI based on proteomics.

外泌体(exosomes)是一类由细胞分泌的直径为30~200 nm的细胞外小囊泡,广泛存在于血液、尿液、唾液和脑脊液等天然生物流体中^[1]^。外泌体带有来自母体细胞的多种蛋白质、核酸、脂质和其他生物活性物质,在细胞间通讯和免疫反应等生理和病理过程中发挥重要作用,因此在临床应用上作为疾病生物标志物和治疗性药物载体具有巨大前景^[2]^。脑脊液(cerebrospinal fluid, CSF)是存在于脑室和蛛网膜下腔的无色液体,通过血脑屏障和脑组织直接相连,能够提供与中枢神经系统相关的健康和疾病信息^[3]^。与脑脊液相比,脑脊液来源外泌体中含有高比例的脑源性蛋白质,能够更好地反映脉络丛和脑白质区域的蛋白质表达情况,而且几种已知的神经退行性疾病生物标志物已经在脑脊液外泌体中被鉴定到,表明脑脊液外泌体在疾病诊断上具有潜在应用价值^[4,5]^。然而,囊泡流式细胞术和纳米颗粒追踪分析的检测结果都表明,脑脊液中的囊泡含量远低于血浆或血清中的囊泡含量^[6]^,因此脑脊液外泌体的分离存在挑战。

截至目前,已经开发了多种用于体液中外泌体分离的技术,例如,基于大小的分离方法包括超速离心(ultracentrifugation)^[7]^、超滤(ultrafiltration)^[8]^、体积排阻色谱(size exclusion chromatography, SEC)^[9]^和场流分级分离(field-flow fractionation)^[10]^,基于电荷的分离方法包括离子交换色谱^[11]^和电泳^[12]^,以及基于聚合物^[13]^或者免疫亲和力^[14]^的方法。其中SEC方法具有保持外泌体完整、产量高和操作简单的优势,并且有研究表明,和商业化的基于负压振荡系统和双耦合谐波振荡系统的外泌体分离系统EXODUS相比,基于SEC方法分离的外泌体纯度更高^[15]^。因此,SEC方法已经被用于分离脑脊液外泌体^[15⇓-17]^。

随着研究的深入,外泌体被进一步分为各类性质不同的亚型,例如尺寸、密度、膜表面受体表达和生物来源等不同的亚型。因为不同亚型的外泌体生物学功能存在差异,对外泌体异质性的研究有助于理解其在生理和病理中的作用机制^[18]^。近几年已经开发了多种方法用于分离外泌体亚群,包括基于大小差异的体积排阻色谱法^[19]^、基于密度差异的密度梯度离心法^[20]^和基于膜表面蛋白表达差异的免疫亲和力方法^[21]^等。本课题组此前发展了二维体积排阻色谱法,分离得到尿液来源的大、中、小3种尺寸外泌体亚群,发现其在翻译后修饰蛋白分子表达谱上存在差异,在大尺寸外泌体中鉴定到了更多与免疫反应过程相关的高表达蛋白,而在中尺寸和小尺寸外泌体中则分别鉴定到更多与生物代谢过程和分子转运过程相关的高表达蛋白^[19]^。Matsumura等^[20]^利用密度梯度离心的方法从肿瘤细胞培养基中分离得到高密度和低密度亚型,其中低密度亚型具有更大的尺寸、更负的表面电位,而且膜表面磷脂酰丝氨酸的表达更高。Matsuda等^[21]^利用抗体功能化珠分别分离得到四跨膜蛋白超家族成员CD9、CD63和CD81阳性的外泌体,发现CD81阳性外泌体和CD9、CD63阳性外泌体在聚糖表达上存在显著不同。目前相关报道侧重于研究血液和尿液等^[18]^,尚未见脑脊液外泌体亚群的相关报道。

创伤性脑损伤(traumatic brain injury, TBI)是由施加到头部的机械力引起的脑组织器质性损伤。全世界每年有5000多万人受到创伤性脑损伤,这一疾病已成为重大的公共卫生问题^[22]^。TBI是全球导致死亡和终身残疾的主要原因之一,同时该疾病还可能引起许多精神退行性疾病,增加幸存者出现慢性行为和神经损伤的风险,影响其生活质量^[23]^。TBI的病理过程复杂,涉及创伤后立即出现的原发性损伤和继发性损伤,可能导致神经元和星形胶质细胞损伤、轴突破坏和炎症,全面了解TBI病理生理过程对于开发治疗干预措施至关重要^[23]^。在液体活检中,外泌体已被报道为TBI潜在的生物标志物之一^[24]^。目前对TBI患者体液中外泌体的研究主要使用血液或唾液样本^[7,25⇓-27]^,而对脑脊液样本的研究较少^[28]^。Manek等^[28]^通过超速离心的方法分离了脑脊液外泌体,并对其进行蛋白质组学研究,与健康对照相比,TBI患者脑脊液外泌体的蛋白质鉴定数量显著升高,这些外泌体可能来源于大脑中的神经元和星形胶质细胞等脑细胞类型。如果能够进一步分离得到不同的脑脊液外泌体亚群并对其进行蛋白质组学鉴定,通过定性和定量分析比较不同亚群间的内含分子,解析它们之间差异化的分子表达,无疑将为研究特定亚型潜在选择性封装的生物标志物提供技术支撑。

针对上述问题,基于本课题组之前开发的血样和尿样外泌体分离的方法^[9,19]^,我们设计了串联体积排阻色谱法用于不同尺寸脑脊液外泌体的分离,并结合纳升级液相色谱-捕集离子淌度飞行时间质谱技术(nanoLC-TIMS-TOF-MS)对创伤性脑损伤患者的外泌体亚群进行蛋白质组学分析,探究亚群间的蛋白质表达差异。本研究分离得到脑脊液外泌体亚群,并从蛋白质组学的角度丰富了对创伤性脑损伤患者脑脊液外泌体的认知,为后续研究疾病中脑脊液外泌体的潜在生物标志物和病理机制提供了方法基础。

## 1 实验部分

### 1.1 仪器、试剂与材料

LC-2010A色谱系统购自日本岛津公司;Multiskan FC酶标仪和NanoDrop 2000C超微量分光光度计购自美国Thermo Fisher Scientific公司;GelDoc XR+IMAGELAB凝胶成像系统购自美国Bio-Rad公司;HT7700 Exalens透射电子显微镜购自日本日立公司;ZetaView PMX 110纳米颗粒追踪分析仪(NTA)购自德国Particle Metrix公司;Concentrator plus真空离心浓缩仪购自德国Eppendorf公司;NanoElute2纳升液相色谱和tims TOF Pro2质谱购自德国Bruker公司。

三(2-羧乙基)膦盐酸盐(tris(2-carboxyethyl)phosphine hydrochloride, TCEP)购自上海泰坦科技股份有限公司;三氟乙酸(trifluoroacetic acid, TFA)和碘乙酰胺(iodoacetamide, IAA)购自美国Sigma-Aldrich公司;质谱级乙腈(acetonitrile, ACN)和甲酸(formic acid, FA)购自德国Merck公司;质谱级胰蛋白酶(trypsin)购自北京生夏蛋白技术有限公司。亲和层析柱空柱管(12 mL)、BCA蛋白浓度测定试剂盒(增强型)、BeyoGel^TM^ Plus PAGE预制胶(Tris-Gly,梯度胶的含量为4%~20%)、一抗稀释液、二抗稀释液、含吐温 20 的三羟甲基氨基甲烷盐酸盐缓冲液(TBST溶液)、QuickBlock封闭液和BeyoECL Star(特超敏ECL化学发光试剂盒)购自上海碧云天生物技术有限公司;4%交联琼脂糖凝胶CL-4B(粒径:45~165 μm)购自上海源叶生物科技有限公司;免疫印迹检测(Western blotting, WB)所用的兔源一抗HSP70、TSG101和CD9购自杭州华安生物技术有限公司,兔源一抗载脂蛋白A1(apolipoprotein A1, ApoA1)和羊源抗兔二抗购自杭州联科生物科技有限公司;SOLAμ^TM^固相萃取孔板购自美国Thermo Fisher Scientific公司;100 kDa超滤管、聚偏二氟乙烯(polyvinylidene fluoride, PVDF)微孔膜和0.22 μm滤膜购自德国EMD Millipore公司。实验用水由Milli-Q超纯水系统制备得到。

### 1.2 脑脊液样品的收集、前处理及伦理声明

2例脑出血患者术后的脑脊液由上海市浦东医院收集,样本信息见表1。通过脑室穿刺引流留取脑脊液约2 mL,样品在4 ℃下以3000 g离心30 min,随后取上清液以10000 g离心30 min。用0.22 μm水系滤膜过滤上清液后,通过100 kDa超滤管(15 mL)将样品浓缩至约400 μL。滤液转移至新的冻存管中,分装冻存于-80 ℃,等待进一步处理。

**表1:** 创伤性脑损伤患者的信息

Index	Age	Gender	Post-operative time for sampling/d
CSF1	79	male	1
CSF2	82	female	2

所有样本采集的志愿者均被告知相关事宜并签署知情同意书。本研究符合国家相关的科研伦理规范,并且取得上海市浦东医院伦理委员会的批准(批准文号:QWJW-12)。

### 1.3 交联琼脂糖凝胶Mini-SEC柱的制备

将4%交联琼脂糖凝胶填料用去离子水配成匀浆,转移至空柱管中,空柱管的底部含有直径与其相匹配的孔径为50 μm的筛板。填料缓慢灌入空柱管,确保柱中没有气泡后在顶部加入孔径为50 μm的筛板,用推杆将柱床压实,柱床为15 mm×5 cm。最后用20 mL的磷酸盐缓冲液(PBS)清洗柱床。

### 1.4 分离纯化脑脊液外泌体亚群

首先用自制的Mini-SEC柱初步分离得到外泌体。将经过前处理的脑脊液样品上样到凝胶柱中,用PBS进行洗脱。每500 μL溶液收集一个馏分,并按照数字顺序编号。收集20个馏分后,用20 mL的PBS清洗柱床,再用于下一个样品的分离。将含有相对较纯外泌体的7~10号馏分合并,并利用100 kDa超滤管(0.5 mL)浓缩至100 μL以内。

浓缩后的馏分进一步通过高效液相体积排阻色谱系统(high performance liquid-size exclusion chromatography, HPL-SEC)进行分离。将KW-804体积排阻柱(填料粒径7 μm,孔径50 nm, 300 mm×8.0 mm,日本Shodex公司)连接至LC-2010A色谱系统上,流动相为PBS,色谱流速为1.0 mL/min,紫外检测器的波长设定为280 nm,柱温设置为25 ℃。依据紫外信号峰收集馏分,再用100 kDa超滤管(0.5 mL)将馏分浓缩至约50 μL。

### 1.5 外泌体的表征

#### 1.5.1 免疫印迹检测

首先,使用BCA蛋白浓度测定试剂盒测定馏分中蛋白质的浓度,采用酶标仪测定560 nm处的吸光度,根据测得结果将样品稀释成相同浓度。取含有10 μg蛋白质的样品加入5×蛋白上样缓冲液,煮沸10 min,利用预制胶在120 V下对不同样品进行分离。采用湿法转膜法,在180 mA下将胶内蛋白转移至PVDF膜上,随后将膜浸泡于QuickBlock封闭液中,室温下摇床上摇晃1 h。将一抗用一抗稀释液按照1∶1000(v/v)的比例稀释,将PVDF膜裁剪后放置于相应的一抗溶液中,4 ℃孵育过夜,随后用TBST溶液洗涤PVDF膜5次,每次10 min。将二抗用二抗稀释液按照1∶3000(v/v)的比例稀释,将孵育过一抗的PVDF膜置于二抗溶液中,室温下孵育2 h,并用TBST溶液洗涤5次。最后在PVDF膜上滴加电化学发光显影液,并于凝胶成像系统中显影拍照。

#### 1.5.2 透射电子显微镜分析

根据BCA蛋白浓度定量的结果将馏分用0.9%氯化钠溶液稀释至约0.02 mg/mL。将5 μL稀释后的样品滴于亲水化处理后的200目铜网上,静置2 min后用毛边滤纸吸去多余液体。随后,滴加5 μL的2%醋酸双氧铀,静置2 min后用毛边滤纸吸去多余染料。最后利用透射电子显微镜在120 kV下观察外泌体形态。

#### 1.5.3 纳米颗粒追踪分析

使用纳米颗粒追踪分析仪检测馏分中外泌体的粒径分布。首先用超纯水和PBS清洗样品池,并用100 nm的聚苯乙烯微球作为标准品校准仪器。随后,用PBS稀释馏分以达到检测窗口所需浓度,用仪器进行检测。

### 1.6 蛋白质组学样品的制备

取浓缩并经BCA蛋白定量后的50 μL不同尺寸外泌体馏分,向其中加入50 μL尿素(12 mol/L),冰上反应30 min后加入终浓度为5 mmol/L的TCEP,室温下反应30 min,再加入终浓度为10 mmol/L的IAA,室温避光反应30 min。加入200 μL的50 mmol/L NH_4_HCO_3_溶液,使尿素浓度降低至2 mol/L。加入终浓度为6 ng/μL的胰蛋白酶,于37 ℃摇床中酶解反应12 h后,加入1% TFA终止酶解。酶解液使用SOLAμ^TM^固相萃取孔板除盐,再利用真空离心浓缩仪干燥。最后用含有2% ACN和0.1% FA的水溶液复溶,储存于-80 ℃。

### 1.7 NanoLC-TIMS-TOF-MS分析

用超微量分光光度计对肽段进行定量。 基于定量结果取100 ng 肽段,采用nanoLC-TIMS-TOF-MS对肽段进行分析。在 300 nL/min的流速下,用Aurora Elite CSI C18 UHPLC色谱柱(15 cm×75 μm, 1.7 μm, 美国Ion Opticks公司)进行分离。流动相A:含有0.1%(v/v)FA的水溶液;流动相B: 含有0.1%(v/v) FA的ACN溶液。采用下述梯度程序进行洗脱分离:0~3 min, 2%B~6%B; 3~16 min, 6%B~22%B; 16~24 min, 22%B~31%B; 24~27 min, 31%B~72%B; 27~30 min, 72%B。柱温设置为60 ℃。

质谱在数据依赖采集-同步累积连续碎裂(DDA-PASEF)模式下运行,在正离子模式下采集*m/z*为100~1700的离子,离子的累积及释放时间为100 ms。一个周期的时间为1.17 s,其中包括一次完整的TIMS扫描和10次 PASEF扫描。在PASEF扫描期间,碰撞能量随离子迁移率线性增加,从1/*K*_0_=0.6 V·s/cm^2^的20 eV增加到1/*K*_0_=1.6 V·s/cm^2^的59 eV(其中*K*_0_是折合离子迁移率)。单电荷前体离子用多边形过滤器排除。用于MS/MS分析的前体离子的强度阈值为2500,目标值为20000,动态排除时间为24 s。其他质谱参数设置如下:离子源类型为ESI,离子传输毛细管电压为4500 V;辅助气体流速为3 L/min;电离温度为180 ℃。

### 1.8 数据分析

对于非标定量(label-free quantification, LFQ)质谱分析,质谱源文件加载到由Biognosys公司开发的SpectroMine软件(版本为4.5.240625.52329)中,数据库为Uniprot的Human Reference Genome(下载时间为2024年3月24日,含20597个蛋白质)。最大漏切位点设置为2。半胱氨酸碘乙酰化为固定修饰,甲硫氨酸氧化和N端乙酰化设置为可变修饰。SpectroMine软件输出的蛋白丰度结果以LFQ强度计算。

蛋白质组学的统计分析均使用OriginPro 2025完成。通过韦恩图显示不同外泌体亚群鉴定到蛋白质的区别。利用FunRich(v3.1.4)对鉴定到的所有蛋白质和Vesiclepedia外泌体数据库(http://microvesicles.org)进行比较^[29]^。对于层次聚类分析,LFQ强度首先使用log_2_变换,并将缺失值填补为##,蛋白质的表达水平通过*Z*-score进行归一化,聚类方法使用Group Average,并以欧氏距离(Euclidean distance)作为聚类簇间的量度。利用ImageGP平台(https://www.bic.ac.cn/BIC/#/)进行主成分分析(principal component analysis, PCA)^[30]^。利用DAVID(Database for Annotation, Visualization and Integrated Discovery)数据库对鉴定到的所有蛋白质进行基因本体论(gene ontology, GO)分析^[31]^。利用Metascape平台(https://metascape.org/)进行通路富集分析,其中对于不同外泌体亚群各自高度表达的基因列表,利用以下本体来源进行分析,包括KEGG Pathway、GO Biological Processes、Reactome Gene Sets、Canonical Pathways、CORUM和WikiPathways^[32]^。通过STRING平台(https://cn.string-db.org/)进行蛋白质-蛋白质相互作用分析^[33]^。

## 2 结果与讨论

### 2.1 脑脊液外泌体亚群的分离

据报道,根据纳米颗粒追踪分析的结果,健康人脑脊液中含有约2×10^8^~7×10^9^particles/mL,包括细胞外囊泡和脂蛋白等^[6]^。而创伤性脑出血患者的脑脊液中存在血液干扰,血液相比于其他生物体液含有高丰度蛋白等更加复杂的生物背景。因此,在脑脊液外泌体的分离过程中需要减少脂蛋白和复杂的生物背景干扰,从而尽可能减小后续对外泌体蛋白检测的影响。对此,我们设计了基于串联体积排阻色谱的脑脊液外泌体亚群分离和纯化方法。整个分离过程如图1a所示,首先,将收集得到的脑脊液通过离心去除细胞和细胞碎片,经超滤浓缩提高样品中外泌体的浓度,并去除一些小蛋白和肽段,接着采用自制Mini-SEC柱分离得到外泌体,去除大量的蛋白质干扰,然后再利用HPL-SEC进一步分离,得到不同尺寸的外泌体。对于单个脑脊液样本,分离纯化得到外泌体亚群的整个过程所用时间仅需1 h左右,该方法具有快速、简单和纯度较高的优势。

**图1:**
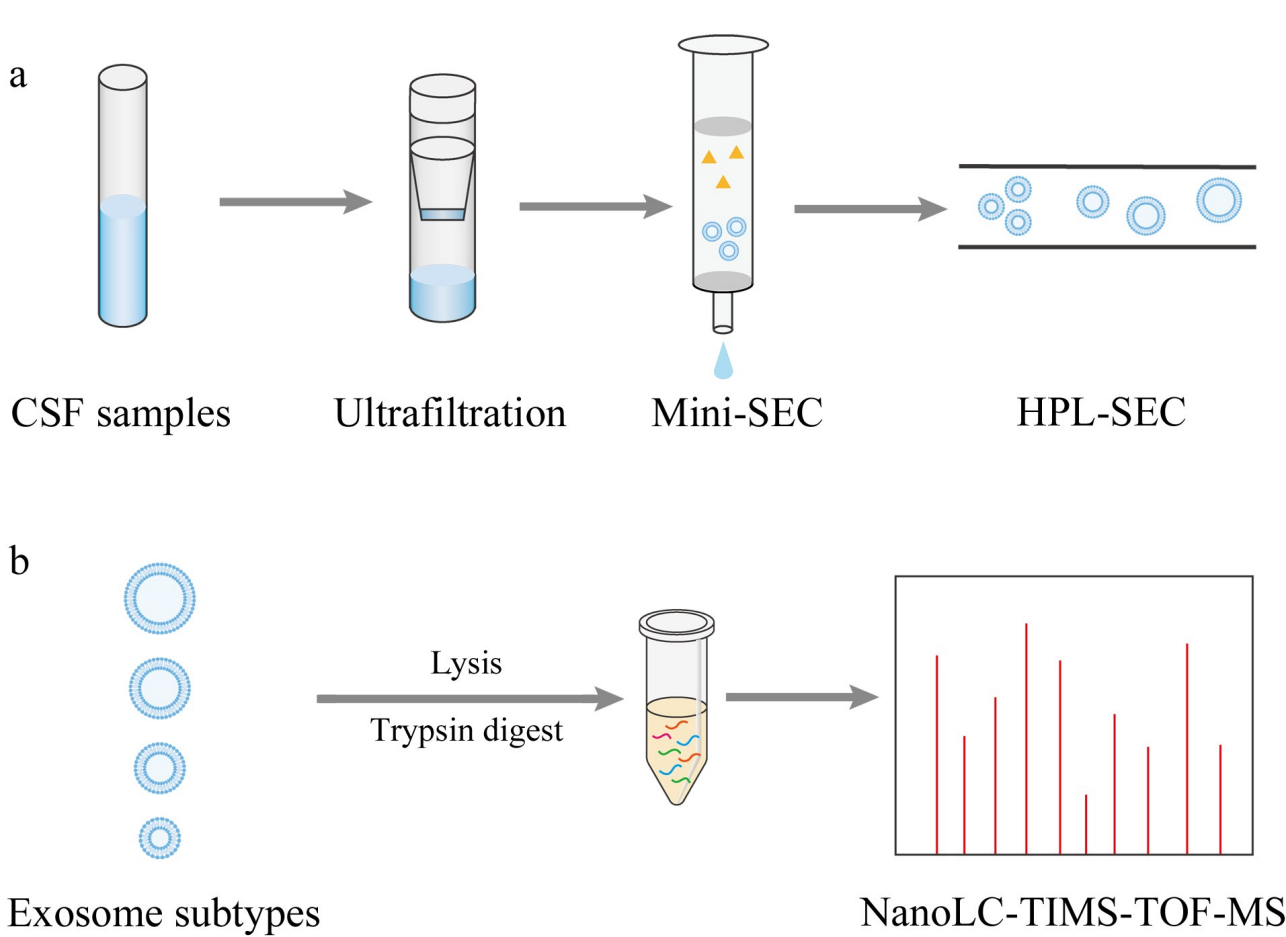
脑脊液来源的不同尺寸的外泌体亚群的(a)分离和(b)蛋白质组学分析流程

第一步,将经过前处理的脑脊液样品用自制Mini-SEC柱分离。首先,对收集到的馏分进行蛋白质含量测定。如图2a所示,最早流出的1~5号馏分的成分是平衡凝胶柱使用的PBS,因此检测不出蛋白质;在7号馏分之后,蛋白质含量逐渐上升,而15号馏分及之后的馏分中蛋白质含量减小。随后,我们对5~15号馏分进行了免疫印迹检测,分析外泌体和常见的脂蛋白ApoA1的表达分布。如图2b所示, 外泌体的生物标志蛋白HSP70、TSG101和CD9存在于7~15号馏分,脂蛋白ApoA1大量存在于11~15号馏分中。为了保证外泌体的产量,同时减少收集得到的外泌体馏分中脂蛋白的污染,7~10号馏分被选择合并作为外泌体馏分,用于后续实验。

**图2:**
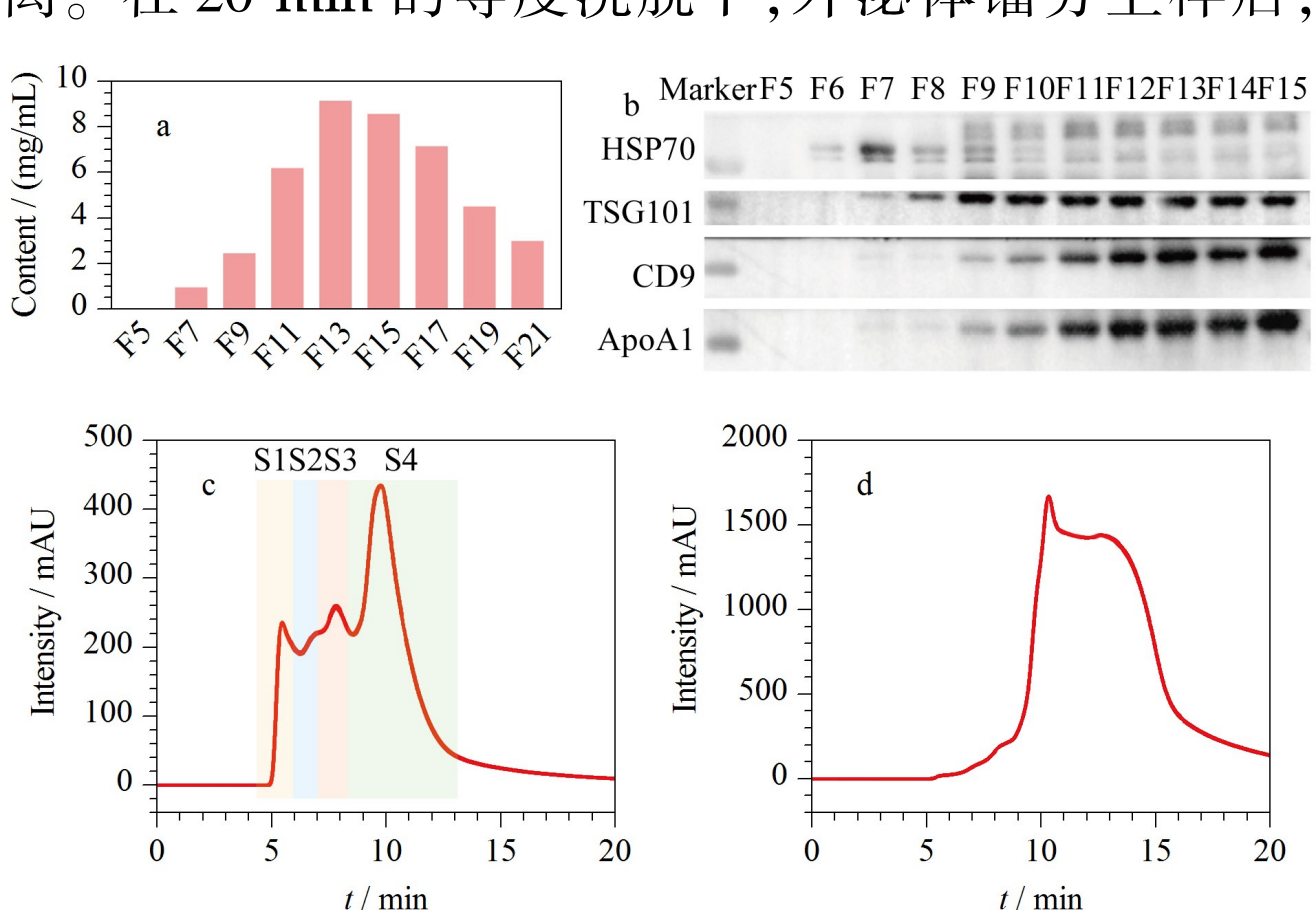
Mini-SEC柱馏分的(a)蛋白质含量和(b)免疫印迹检测 图像;Mini-SEC柱(c)7~10号馏分和(d)11~14号馏分分别合并后的HPL-SEC色谱图

第二步,将外泌体馏分用HPL-SEC进一步分离。在20 min的等度洗脱下,外泌体馏分上样后,获得的色谱图在保留时间5~13 min出现了4个色谱峰(图2c),分别标记为S1、S2、S3和S4。将Mini-SEC柱收集的11~14号馏分合并后,同样经HPL-SEC分离(图2d),保留时间为10~15 min的信号强度远远高于外泌体馏分色谱图(图2c)的信号强度,结合图2b的免疫印迹检测结果,说明11~14号馏分中含有大量的蛋白质干扰。随后,利用纳米颗粒追踪分析仪测得外泌体含量,并用BCA蛋白浓度测定试剂盒测定样品的蛋白质含量。以外泌体颗粒的数量与总蛋白质含量的比值作为评价纯度的指标^[34]^,得到超滤浓缩以及2次SEC处理后的外泌体纯度如表2所示。与仅超滤浓缩处理的样品相比,Mini-SEC柱处理后的外泌体纯度提高了约12倍。进一步用HPL-SEC分离得到的4个馏分中,S1馏分的外泌体纯度最高,同时,S4馏分的外泌体纯度虽然降低,但是仍比仅超滤浓缩处理的样品纯度高,因此4个馏分均被用于后续表征实验。上述结果进一步证实了Mini-SEC柱去除了大量蛋白质的干扰。

**表2:** 脑脊液经过超滤和2次体积排阻色谱处理后得到的样品的外泌体纯度

Samples/process	Exosome content/(10^10^ particles/mL)	Protein content/(mg/mL)	Particle-to-protein ratio/(10^10^ particles/mg)
Concentrated CSF sample/ultrafiltration	130	139	0.935
Fractions 7-10 mixture/Mini-SEC	150	13.1	11.5
Fraction S1/Mini-SEC and HPL-SEC	37.4	0.621	60.2
Fraction S2/Mini-SEC and HPL-SEC	47.6	2.33	20.4
Fraction S3/Mini-SEC and HPL-SEC	39.1	2.00	19.6
Fraction S4/Mini-SEC and HPL-SEC	51.0	7.70	6.62

### 2.2 脑脊液外泌体亚群的表征

我们运用多种手段表征了外泌体馏分经HPL-SEC分离得到的4个亚组分。4个馏分的TEM图像中都能观察到囊泡状的纳米颗粒(图3a),且纳米颗粒的尺寸逐渐减小。进一步用WB实验验证外泌体的获得,不同馏分取相同质量的蛋白质用常见的外泌体生物标志物进行表征。如图3b所示,在S1~S3馏分中,HSP70高表达,TSG101和CD9低表达,其中S2馏分的HSP70表达量最高,而S4馏分相反,TSG101和CD9高表达,HSP70低表达,说明不同尺寸的外泌体表面所表达的抗原可能存在差异。纳米颗粒追踪分析结果显示(图3c), 4个馏分的粒径主要分布在50~200 nm,与之前研究报道的外泌体粒径一致^[19]^。综上可见,串联体积排阻色谱方法实现了不同尺寸外泌体亚群的分离。

**图3:**
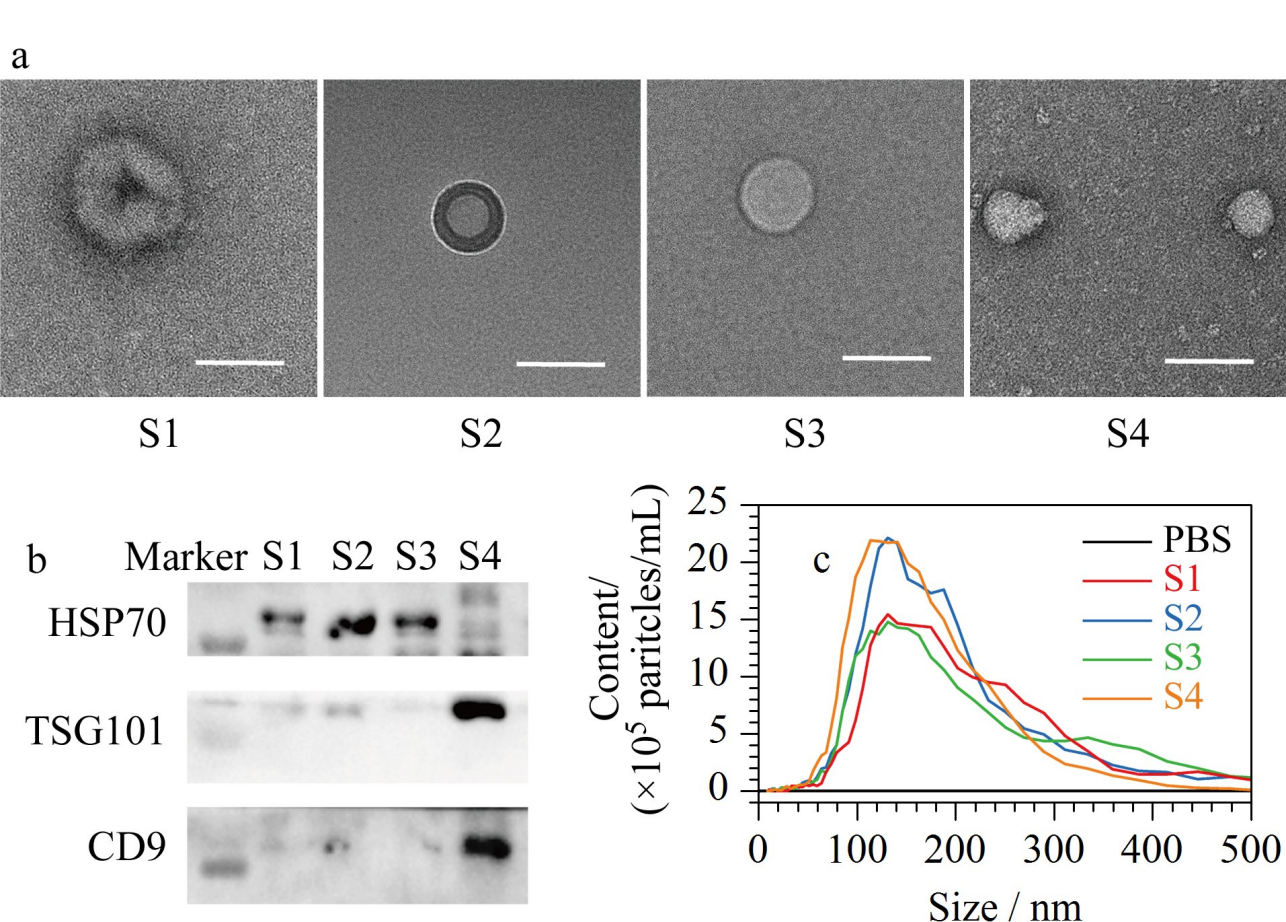
脑脊液外泌体亚群的表征

### 2.3 外泌体亚群的蛋白质组学分析

目前,质谱已经被广泛地用于分析复杂生物样品中的数千种蛋白质。为了分析不同大小外泌体亚群的蛋白质差异,我们以2例脑出血患者术后的脑脊液为样本,利用串联体积排阻色谱法分别处理,再将分离得到的4种尺寸的外泌体亚群(S1~S4)进行蛋白质提取与酶解,并利用nanoLC-TIMS-TOF-MS进行分析,实验流程如图1b所示。质谱结果分析显示,2个样本共鉴定到739个蛋白质,其中高达79%的蛋白质与Vesiclepedia外泌体蛋白数据库相匹配^[35]^,且有72个蛋白质与数据库中按鉴定次数排序的前100个蛋白质相匹配(图4a和4b)。韦恩图(图4c)显示样本1的S1~S4亚群中分别鉴定到528、431、433和400个蛋白质,样本2的S1~S4亚群中分别鉴定到411、397、377和383个蛋白质。4个亚群中S1亚群的蛋白质鉴定量最高,且同一样本的不同亚群间共同蛋白质占比约为40%,说明不同外泌体亚群鉴定蛋白质存在差异。

**图4:**
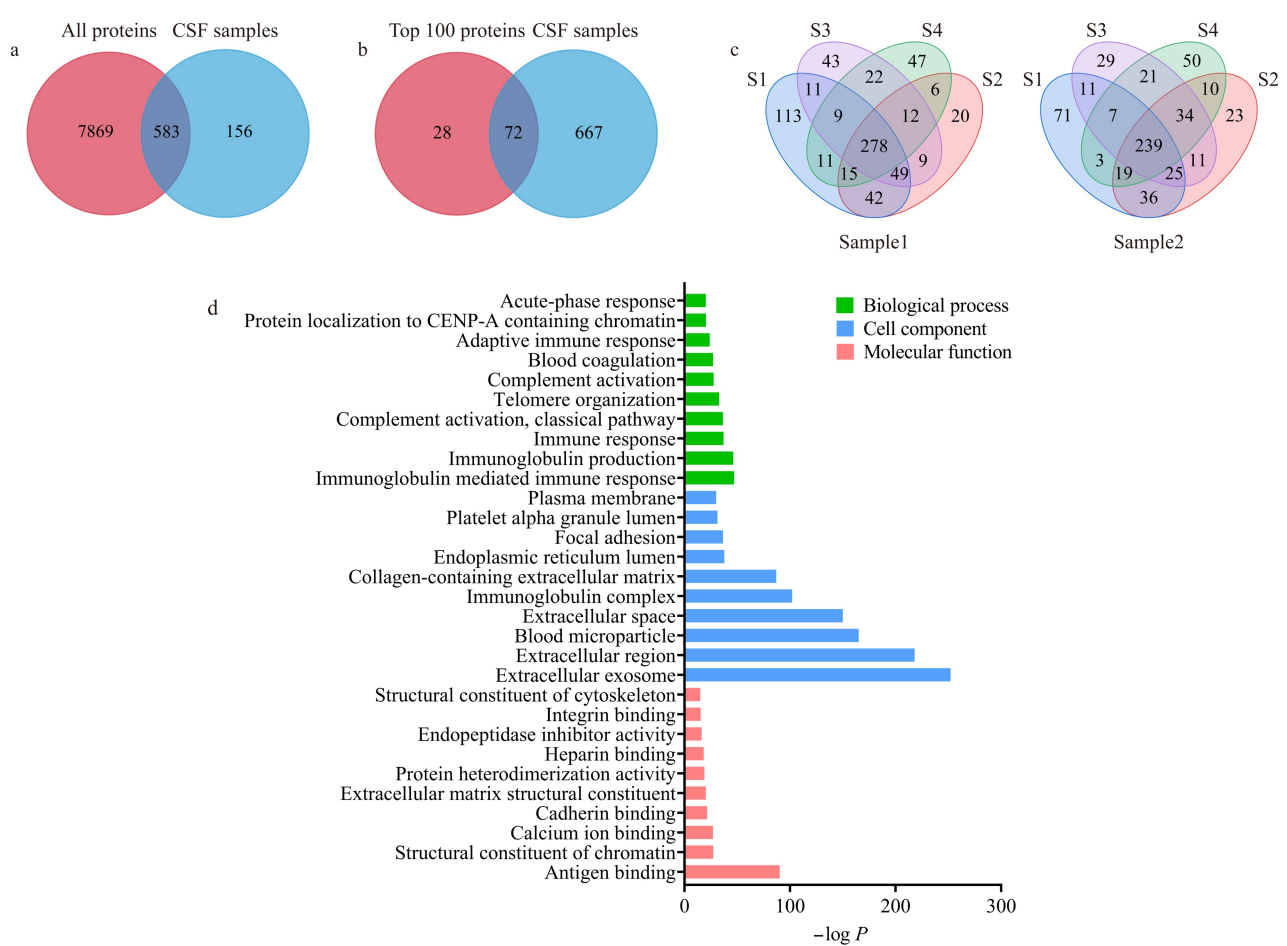
鉴定到的脑脊液外泌体蛋白质与Vesiclepedia外泌体数据库中(a)所有蛋白质和(b)按鉴定次数排序的前100个蛋白质的对比;(c)两个脑脊液样本外泌体亚群中鉴定到蛋白质的韦恩图;(d)脑脊液外泌体蛋白质的基因本体论分析

对样本中鉴定到的所有蛋白质进行GO分析,将生物学过程(biological process)、细胞组分(cell component)和分子功能(molecular function)中按顺序列出前10名最具有统计学意义的富集功能通路(图4d)。基因富集分析显示,生物学过程中排名前3的结果都与免疫相关,特别是免疫球蛋白介导的免疫反应,同时排名前10中包括“Blood coagulation”,这是因为使用的脑脊液样品来源于创伤性脑损伤患者,与预期相符。细胞组分中排名第一的是“Extracellular exosome”,反映鉴定到的蛋白质是封装进外泌体的。分子功能分析显示排名靠前的涉及抗原、钙离子、钙黏蛋白、肝素和整合素结合等,这些丰富的分子功能与细胞信号转导、凝血过程和免疫反应等生物过程密切相关。

为了进一步探究不同尺寸的外泌体亚群所含蛋白质的差异性,我们利用非标定量质谱的方法对鉴定到的蛋白质进行定量分析,蛋白质的丰度用LFQ强度来代表,经过log_2_变换和归一化后用于后续分析。如图5a所示,蛋白质的表达水平在不同亚群之间展现出了明显的差异,其中来自同一个尺寸亚群的样品首先聚类,因为S2和S3亚群在色谱保留时间上相距较近,尺寸差异较小,因此在更高簇上先聚类。同时我们也发现,来自两个样本的S1亚群存在相对较大的差异,我们推测S1亚群可能更能代表不同样本的差异性,未来对此进一步研究可能有利于实现不同患者精准化治疗方案的制定。基于非标定量结果的主成分分析显示(图5b),不同亚群之间能够较好地分隔。如图5c所示,皮尔逊相关性系数(Pearson’s correlation coefficients)分析表明,对于S2、S3和S4亚群,同一亚群中不同样品间系数超过0.75,不同亚群间样品的系数低于0.7,但是对于S1亚群,两个样品间的系数仅0.55,说明差异较大,这与前述层次聚类分析结果一致,进一步证实了在创伤性脑损伤患者的脑脊液外泌体中蛋白质是差异分布的。

**图5:**
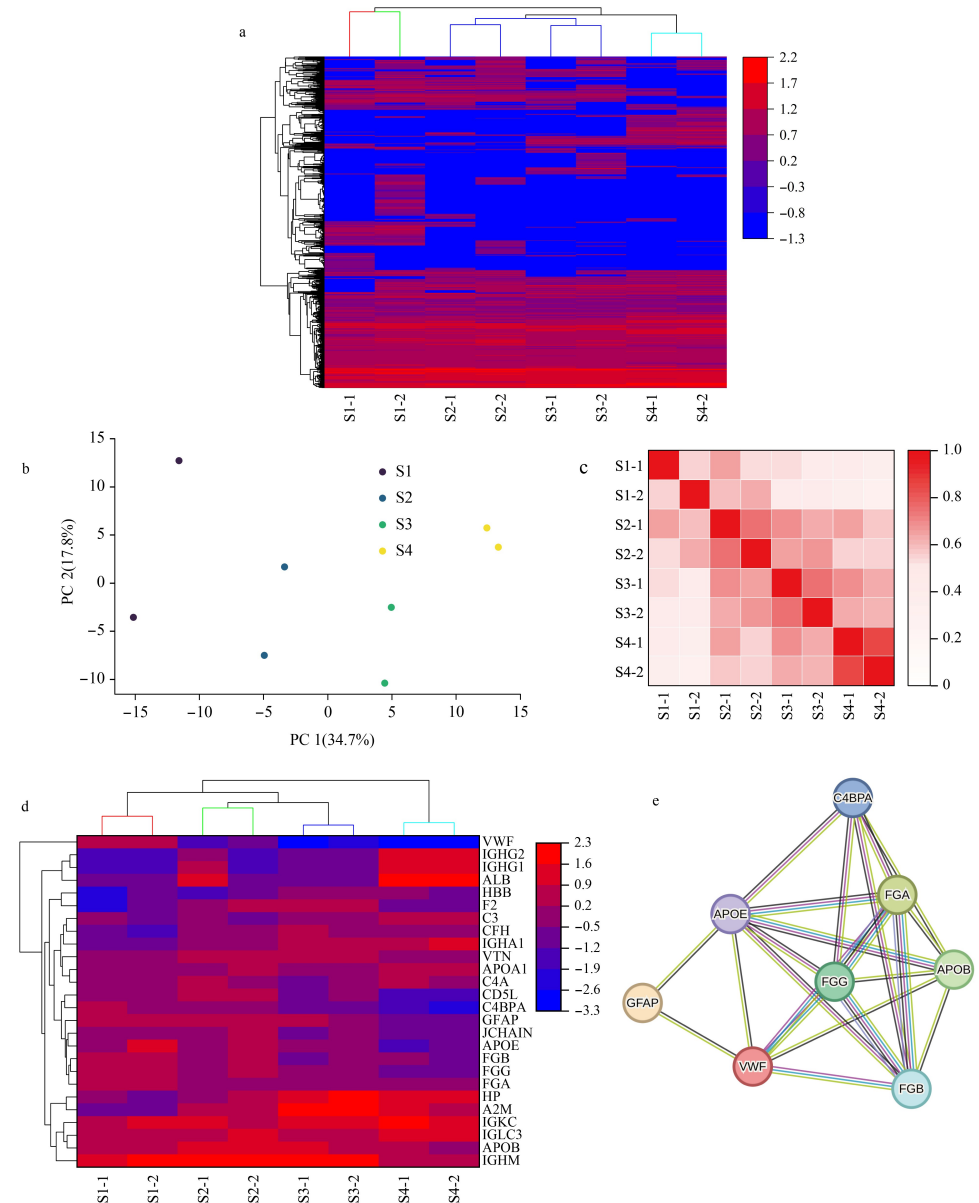
脑脊液外泌体亚群的蛋白质组学分析

神经细胞黏附分子L1(L1CAM)是在神经元上表达的跨膜蛋白,传统上被认为是神经元来源外泌体(neuron-derived extracellular vesicles, NDEVs)的标志^[36]^,但是Norman等^[17]^的研究证明L1CAM与人脑脊液来源的外泌体不相关,不能作为可靠的NDEVs的生物标志物。在我们的研究中,两个样本的4个馏分中均未检测到L1CAM,这一结果说明L1CAM可能与脑脊液外泌体没有强烈关联,与Norman等^[17]^的研究结果一致。

我们对不同尺寸外泌体亚群各自高表达的蛋白质进行了分析。首先筛选得到每个样本4个外泌体亚群的LFQ强度排名前10的蛋白质,随后合并两个样本中同一亚群的蛋白质作为该亚群的高表达蛋白质,再利用Metascape平台进行通路富集分析。如表3所示,S1亚群富集通路包括维生素B12代谢和蛋白质分解代谢过程的调控,S2亚群富集通路包括Scavenger受体对配体的结合和吸收、血浆中清除血红素和炎症反应,S3亚群特异富集的通路包括补体和凝血级联反应和急性期反应,S4亚群特异富集的通路包括翻译后蛋白磷酸化。高表达蛋白质在外泌体亚群的差异表达如图5d所示。热图可视化的结果显示,免疫球蛋白相关的蛋白质(IGKC、IGLC3和IGHM)和载脂蛋白B(APOB)在所有外泌体亚群中都是高表达的。与其他外泌体亚群相比,在S1亚群中,血管性血友病因子(von Willebrand factor, VWF)是高表达的,该蛋白与止血过程有关,而在S4亚群中,免疫球蛋白重链的恒定区(IGHG1和IGHG2)和人血清白蛋白(ALB)高表达。据报道,胶质纤维酸性蛋白(GFAP)几乎仅在中枢神经系统的星形胶质细胞中表达,当星形胶质细胞的细胞骨架受损时,GFAP会被激活释放,可能可以作为一种潜在的TBI生物标志物^[37]^。GFAP在S1、S2、S3亚群中均是表达排名前10的蛋白质,在S4亚群中表达相对较低。此外,我们进一步对S1亚群的高表达蛋白质利用STRING进行蛋白质-蛋白质相互作用分析,以功能和物理蛋白质关联绘制网络,如图5e所示,图中线条的颜色表示相互作用的类型,当设置最低交互分数为中等置信度(0.400)时,结果显示存在着多种蛋白质间相互作用,反应组通路富集分析结果包括“GRB2和SOS为整合素提供与MAPK信号通路的联系”“p130Cas为整合素提供与MAPK信号通路的联系”“内源配体对TLR的调控”等。综上所述,上述结果初步揭示了脑脊液来源的外泌体亚群的蛋白质表达存在异质性,不同尺寸的外泌体亚群可能有特定的生物学功能。

**表3:** 两个样品的外泌体亚群各自高表达蛋白质的通路和过程富集分析

Pathway description	Number of genes			
	S1	S2	S3	S4
Vitamin B12 metabolism	5		3	
Regulation of protein catabolic process	3			
Binding and uptake of ligands by		5	3	3
scavenger receptors				
Scavenging of heme from plasma		3		
Inflammatory response		3		
Complement and coagulation cascades			4	3
Acute-phase response			3	
Post-translational protein phosphorylation				4

## 3 结论

本研究设计了串联体积排阻色谱法,减少了干扰蛋白质,分离得到不同尺寸的脑脊液来源的4个外泌体亚群。结合nanoLC-TIMS-TOF-MS,以创伤性脑损伤患者为研究对象对外泌体亚群进行蛋白质组学分析,结果表明分离得到的外泌体与细胞信号转导、凝血过程和免疫反应等生物过程密切相关。此外,外泌体亚群的蛋白质表达存在异质性,特别是大尺寸的亚群在不同样本间存在较大的差异,可能有助于实现不同患者精准化治疗方案的制定。受限于健康对照志愿者脑脊液样本的获取,目前对脑脊液来源外泌体的研究仍然有限,利用本研究开发的方法进行进一步研究可能能够为创伤性脑损伤疾病脑脊液来源外泌体的作用机制带来新的认知。
